# Variability in Susceptibility to Type I Interferon Response and Subgenomic RNA Accumulation Between Clinical Isolates of Dengue and Zika Virus From Oaxaca Mexico Correlate With Replication Efficiency in Human Cells and Disease Severity

**DOI:** 10.3389/fcimb.2022.890750

**Published:** 2022-06-21

**Authors:** Tannya Karen Castro-Jiménez, Laura Cristina Gómez-Legorreta, Laura Alejandra López-Campa, Valeria Martínez-Torres, Marcos Alvarado-Silva, Araceli Posadas-Mondragón, Nallely Díaz-Lima, Hilda Arcelia Angulo-Mendez, Nancy R. Mejía-Domínguez, Felipe Vaca-Paniagua, Federico Ávila-Moreno, Julio García-Cordero, Leticia Cedillo-Barrón, Sergio Roberto Aguilar-Ruíz, José Bustos-Arriaga

**Affiliations:** ^1^ Laboratorio de Biología Molecular e Inmunología de arbovirus, Unidad de Biomedicina, Facultad de Estudios Superiores Iztacala, Universidad Nacional Autónoma de México, Tlalnepantla, Mexico; ^2^ OaxacaLab Laboratorio de análisis Clínicos, Oaxaca, Mexico; ^3^ Red de Apoyo a la Investigación, Universidad Nacional Autónoma de México e Instituto Nacional de Ciencias Médicas y Nutrición Salvador Zubirán, Mexico City, Mexico; ^4^ Unidad de Biomedicina, Facultad de Estudios Superiores Iztacala, Universidad Nacional Autónoma de México, Tlalnepantla, Mexico; ^5^ Departamento de Biomedicina Molecular, Centro de Investigación y de Estudios Avanzados del Instituto Politécnico Nacional, Ciudad de México, Mexico; ^6^ Departamento de Biomedicina Experimental, Facultad de Medicina y Cirugía de la Universidad Autónoma ‘Benito Juárez’ de Oaxaca, Oaxaca, Mexico

**Keywords:** dengue, Zika, interferons, isolates, sfRNA

## Abstract

Dengue and Zika viruses cocirculate annually in endemic areas of Mexico, causing outbreaks of different magnitude and severity every year, suggesting a continuous selection of *Flavivirus* variants with variable phenotypes of transmissibility and virulence. To evaluate if *Flavivirus* variants with different phenotypes cocirculate during outbreaks, we isolated dengue and Zika viruses from blood samples of febrile patients from Oaxaca City during the 2016 and 2019 epidemic years. We compared their replication kinetics in human cells, susceptibility to type I interferon antiviral response, and the accumulation of subgenomic RNA on infected cells. We observed correlations between type I interferon susceptibility and subgenomic RNA accumulation, with high hematocrit percentage and thrombocytopenia. Our results suggest that *Flaviviruses* that cocirculate in Oaxaca, Mexico, have variable sensitivity to the antiviral activity of type I interferons, and this phenotypic trait correlates with the severity of the disease.

## Introduction

Dengue virus (DENV) and Zika virus (ZIKV) are members of the *Flaviviridae* family and the *Flavivirus* genus. Their mature viral particle consists of genomic RNA on an imperfect icosahedral capsid and an envelope covered with 90 homodimers of the envelope protein (Therkelsen et al., 2018). To date, four serotypes of DENV (DENV1, DENV2, DENV3, and DENV4) and three genotypes of ZIKV (West African, East African, and Asian) have been identified. In Mexico, the four serotypes of DENV and mainly the Asian genotype of Zika cocirculate in most endemic states (Thézé et al., 2018). Like other RNA viruses, DENV and ZIKV are in constant evolution and have high adaptation capabilities. *Flavivirus* variants can be selected by evolutionary pressures like the immune response of the host, resulting in the potential emergence of *Flavivirus* variants with high transmissible or pathogenic potential (Lambrechts et al., 2012; Pollett et al., 2018; Xia et al., 2018; Aubry et al., 2021; de Matos et al., 2021). Although it has been reported that the variability of *Flaviviruses* is comparable to that of other RNA viruses like the human immunodeficiency virus and poliovirus (Jin et al., 2011), phenotypic characterization of selected variants is limited. Some studies have shown that variability in structural proteins might influence the neutralization capability of DENV strains by antibodies from naturally infected and vaccinated individuals (Wahala et al., 2010; Brien et al., 2010; Messer et al., 2012; Arimoto et al., 2015; Katzelnick et al., 2015; Forshey et al., 2016; Gallichotte et al., 2018; Bell et al., 2019; Chen et al., 2020; Martinez et al., 2020). This antigenic variation could explain the documented reinfections with homotypic serotypes of DENV, contributing to rising concerns about incomplete long-term protective immunity to reinfection or reduced vaccine efficacy (Waggoner et al., 2016; Juraska et al., 2018).

Most of the evidence addressing *Flavivirus* variability is focused on neutralization and cross-reactivity among DENV serotypes and strains. However, variability in the sequence of non-structural proteins or the untranslated regions of the viral genome could impact transmissibility or virulence. There is evidence that regions of the viral genome that encode non-structural proteins present substantial variability (Pollett et al., 2018). These differences could lead to critical phenotypic changes since these non-structural proteins are critical to the replication cycle, contain potential T cell epitopes, and have innate immune evasion capabilities (Leung et al., 2008; Rastogi et al., 2016; Tian et al., 2019; Fanunza et al., 2021). Additionally, there is evidence of highly variable regions in the 3′UTR; variability in this region of the gRNA could impact the secondary and tertiary structures that are critical for the accumulation of subgenomic flaviviral RNAs (sfRNAs), which are small RNA products of the incomplete degradation of the gRNA by host 5′–3′ exonuclease XRN1, which have been associated with pathogenesis and type I interferon evasion (Pijlman et al., 2008).

Several efforts have been made to understand the variables that influence the transmissibility and virulence of DENV and ZIKV; the available evidence suggests that a combination of host susceptibility, vector transmissibility, virus variability, and ecological factors can influence the intensity of epidemic outbreaks and severity of the clinical manifestations (Rico-Hesse et al., 1997; OhAinle et al., 2011; Lambrechts et al., 2012; Tabachnick, 2016; Fontaine et al., 2018; Aubry et al., 2021). However, the variability of cocirculating flaviviruses is typically determined by comparing the genome sequence instead of the phenotype. It has been suggested that some strains of DENV and ZIKV could be associated with enhanced severity of outbreaks or epidemiological replacements, but the characterization of phenotypes that could explain these differences in transmissibility or virulence between *Flavivirus* variants is rarely explored (Zhang et al., 2005; De Castro et al., 2013; Xia et al., 2018; Aubry et al., 2021; Inizan et al., 2021).

Variability in the immune evasion of the type I interferons response could influence the pathogenic potential of circulating flaviviruses; Manokaran et al. demonstrated that two different clades of DENV2 induced different transcription levels of the *ifnβ* gene, and this difference correlated with the sfRNA accumulation in infected cells (Manokaran et al., 2015). In this study, we isolated cocirculating DENV and ZIKV from Oaxaca City during the 2016 and 2019 outbreaks and compared the replication kinetics in human cells, their ability to evade the antiviral response of type I interferons, and the accumulation of sfRNAs in infected human cells. We observed that cocirculating isolates of DENV and ZIKV presented different phenotypes of evasion and replication. Additionally, we observed a correlation between the replication and evasion phenotypes of DENV isolates with high hematocrit percentage and thrombocytopenia.

## Materials and Methods

### Ethics Statement

Thirty 30 blood samples from donors from Oaxaca City were collected. These samples were tested by a rapid immunochromatography for dengue NS1 viral antigen and DENV and ZIKV IgM and IgG antibodies. Twelve samples were from 2016 and eighteen from 2019. Additionally, all the samples were analyzed by hematic biometry. Participants provided signed informed consent, all samples were treated anonymously, and no identifiable information was collected. The protocols were approved by UNAM and UABJO ethics committees. The obtained biometric information and lab test results of the samples from a virus isolated are listed in **Table 1**.

**Table 1:** Donor information.

	Oax-2016-1	Oax-2016-2	Oax-2016-3	Oax-2016-4	Oax-2016-5	Oax-2016-6	Oax-2019-1	Oax-2019-2	Oax-2019-3	Oax-2019-4
**Gender**	F	M	F	M	M	M	M	F	F	F
**Age (years)**	35	52	36	20	14	15	56	24	18	31
**DENV NS1**	(−)	(+)	(+)	(+)	(+)	(+)	(−)	(+)	(−)	(−)
**DENV IgM**	(−)	(−)	(+)	(+)	(−)	(+)	(−)	(−)	(−)	(−)
**DENV IgG**	(−)	(−)	(+)	(−)	(−)	(+)	(−)	(−)	(−)	(+)
**ZIKV IgM**	(+)	(−)	(−)	(−)	(−)	(−)	(+)	(−)	(+)	(+)
**ZIKV IgG**	(−)	(−)	(−)	(−)	(−)	(−)	(−)	(−)	(−)	(−)
**CHKV IgM**	(−)	(−)	(−)	(−)	(−)	(−)	(−)	(−)	(−)	(−)
**CHIKV IgG**	(−)	(−)	(−)	(−)	(−)	(−)	(−)	(−)	(−)	(−)
**Hgb (g/dl)**	**12.1**	17.5	15.3	16.1	**17.7**	16.4	15.1	15	12.8	13.9
**Hct (%)**	45.7	**51.9**	49	47.9	**52.9**	47.9	43.5	47.8	39.3	38.9
**RBC (×10^12^/L)**	**4.24**	**5.69**	4.94	**5.64**	**6.23**	5.46	5.5	**5.34**	**4.27**	4.55
**RDW (%)**	13.7	12.8	12.8	13.2	12.7	12.5	**14.2**	12.5	12.8	12.7
**MCV (fl)**	92.69	91.21	92.51	86.88	84.91	87.73	87.09	89.51	91.1	95.6
**MCH (pg)**	28.54	30.76	30.97	28.55	28.41	30.04	**27.45**	28.09	29.98	30.55
**MCHC (g/L)**	307.9	337.2	309.7	328.6	335.6	342.4	**315.2**	**313.8**	329	**319.5**
**PLT (×10^9^/L)**	**148**	**66**	**78**	**123**	**27**	**89**	296	**126**	297	330
**MPT (fl)**	8.5	10.1	**12.3**	10.7	10.4	10.4	8.8	8.1	9.8	10.2
**WBC (10^9^/L)**	8.01	**3.27**	2.02	**2.27**	3.42	6.85	7.94	**3.1**	6.29	6.36
**Neutrophils (%)**	**86.3**	63.3	**42.1**	63.9	**25.4**	**81.3**	**83.2**	68.3	57	**75.3**
**Eosinophils (%)**	0.6	0	0.5	**4**	0.3	0.1	0.1	0.47	1.7	0
**Basophils (%)**	0.2	0.3	0.5	0.4	0.6	0.1	0.1	0.94	0.2	0.2
**Monocytes (%)**	5.7	**17.1**	**10.9**	**13.2**	**26.9**	**9.2**	**9.3**	**10.04**	7.2	**11.6**
**Lymphocytes (%)**	**7.2**	**19.3**	**46**	**18.5**	**46.8**	**9.3**	**7.3**	20.2	33.9	**12.9**
**Bands (%)**	0	0	0	0	0	**4**	**4**	0	1	**6**
**Neutrophils (×10^9^/L)**	6.91	2.07	**0.85**	**1.45**	**0.87**	5.57	6.61	2.12	3.58	4.79
**Eosinophils (×10^9^/L)**	0.05	**0**	**0.01**	0.09	**0.01**	**0.01**	**0.01**	**0.01**	0.11	**0**
**Basophils (×10^9^/L)**	0.02	**0.01**	**0.01**	0.01	0.02	**0.01**	**0.01**	0.03	0.01	**0.01**
**Monocytes (×10^9^/L)**	0.46	0.56	0.22	0.3	**0.92**	0.63	0.74	0.31	0.45	0.74
**Lymphocytes (×10^9^/L)**	**0.58**	**0.63**	**0.93**	**0.42**	1.6	**0.64**	0.58	**0.63**	2.13	**0.82**
**Bands (×10^9^/L)**	0	0	0	0	0	0.27	0.32	0	0.06	0.38

Biometric data, results of the rapid immunochromatography test, and hematic biometry data are listed. All the laboratory tests were performed on the original blood sample; parameters out of the reference limits are highlighted in bold font. Samples are labeled with the Flavivirus isolate code name.

RBC, red blood cells; WBC, white blood cells; Hgb, hemoglobin; Hct, hematocrit; RBC, red blood cell count; RDW, red cell distribution width; MCV, mean cell volume; MCH, mean corpuscular hemoglobin; MCHC, mean corpuscular hemoglobin concentration; WBC, white blood cell count; PLT, platelet count; MPV, mean platelet volume.

### Cell Cultures

Vero cells were cultured in Roswell Park Memorial Institute (RPMI) media pH 7.8 (Biowest, Nuaillé, France) supplemented with 5% fetal bovine serum (FBS) (Biowest) and 1% of antibiotic-antimycotic solution (Biowest). HFF-1 was cultured with Dulbecco’s modified Eagle’s medium (DMEM) media (Biowest) supplemented with 10% FBS (Biowest) and 1% of antibiotic-antimycotic solution (Biowest). U937-DC-SIGN cells were cultured in RPMI media (Biowest) supplemented with 10% FBS (Biowest) and 1% of antibiotic-antimycotic solution (Biowest). All cell lines were incubated in a CO_2_ atmosphere at 37°C.

### *Flavivirus* Isolation

Blind passages of the sera samples were realized; confluent monolayers of Vero cells were inoculated with diluted sera (1:40) and incubated in a CO_2_ atmosphere at 37°C for 7 days or until observation of cytopathic effect (CPE) in at least 30% of the monolayer. Supernatants were collected and cleared by centrifugation at 2,000 rpm for 5 min at 4°C. For cryopreservation, 1/10 of SPG stabilizer (2.18 mM of sucrose (Sigma-Aldrich, St. Louis, MO, USA), 38 mM of monobasic K_2_HPO_4_ (Sigma-Aldrich), 72 mM of dibasic K_2_HPO_4_ (Sigma-Aldrich), and 60 mM of l-glutamic acid (Sigma-Aldrich) were added, and fractionated supernatants were stored at −80°C until further use. For all the experiments, no more than ten passages were used.

Intracellular immunofluorescence was used to confirm the presence of infectious virus during the blind passages; Vero or HFF-1 cells were seeded on glass coverslips (Bellco, Vineland, NJ, USA). After 24 h, the culture medium was removed, and monolayers were inoculated with 100 µl of the supernatant of the blind passage and incubated in a CO_2_ atmosphere at 37°C for 48 h. Monolayers were fixed with 4% *p*-formaldehyde solution in phosphate-buffered saline (PBS) (Sigma-Aldrich) for 20 min at room temperature; cells were then permeabilized with 0.1% Triton X-100 in PBS. The cell monolayer was incubated for 60 min with a mouse anti-pan-*flavivirus* 4G2 antibody (kindly donated by Dr. Stephen Whitehead, LIV, NIH, MD, USA), followed by Alexa Fluor 546-conjugated secondary antibody anti-mouse IgG H+L (Invitrogen, Carlsbad, CA, USA). Non-infected monolayers were used as negative controls. Finally, the nucleus was labeled with DAPI (1 µg/mL) (Invitrogen) in PBS for 10 min, and the slides were mounted with Vectashield (Vector, Burlingame, CA, USA). The images were captured in a confocal microscope (Leica SP8; Leica Biosystems, Wetzlar, Germany).

### Virus Titration

Viruses were titrated using a plaque-forming assay technique using Vero cells; 10-fold serial dilutions of cryopreserved virus preparations or cell supernatants were used to infect the confluent monolayers of Vero cells in 24-well plates. After incubation at 37°C for 1 h, the infected cells were overlaid with RPMI with 1% methylcellulose (Sigma-Aldrich), 2% FBS (Biowest), and 2 mM of l-glutamine (Biowest). After 5 days, the monolayers were washed with PBS, fixed and permeabilized with 80% ice-cold methanol for 15 min, and then blocked with 5% low fat powdered milk diluted in PBS. Plaques were immunostained with a mouse anti-pan-flavivirus 4G2 antibody (kindly donated by Dr. Stephen Whitehead, LIV, NIH, MD, USA) and a peroxidase-conjugated secondary antibody (KPL, Gaithersburg, MD, USA). Finally, plaques were developed with peroxidase substrate (KPL) and counted manually; titers are expressed in PFU/mL.

### Virus Typification by RT-PCR

Vero cells at 1 multiplicity of infection (MOI) were infected with every *Flavivirus* isolate, and at 3 days post-infection (dpi), RNA was extracted using miRNeasy kit (Qiagen, Hilden, Germany) following the manufacturer’s recommendations. RNA was quantified in a nano spectrophotometer (IMPLEN, Westlake Village, CA, USA) after extraction, and 5 μg of total RNA was used to retrotranscribe cDNA. For DENV typification, the nested PCR protocol developed by Lanciotti et al. (Lanciotti et al., 1992) was adapted; briefly, the RNA was retrotranscribed using the dengue serocomplex consensus oligonucleotide D2 with recombinant Moloney Murine Leukemia Virus Retrotranscriptase (Thermo, Wilmington, DE, USA) following the manufacturer’s recommendations. cDNA measuring 1.8 to 3 μg was used for the following PCR, and the serocomplex consensus reaction using D1 and D2 oligonucleotides was performed. Finally, for DENV typing, D2 oligonucleotide was replaced with serotype-specific oligonucleotides, and the PCR products were resolved in 4% agarose gels. For ZIKV identification, 5 μg of total RNA was retrotranscribed with random primers (Thermo), and a fragment of 760 bp was amplified in the NS5 region of the gRNA using the oligonucleotides ZikV9113Fwd TTYGAAGCCCTTGGATTCTT and ZikV9872Rev CYCGGCCAATCAGTTCATC and the protocol designed by Díaz-Quiñonez et al. (Díaz-Quiñonez et al., 2016). All PCRs were performed using the GoTaq master mix kit (Promega, Madison, WI, USA).

### Replication of *Flavivirus* Isolates in Human Cell Lines

Human dermal fibroblast HFF-1 cells (ATCC SCRC-1041) and U937-DC-SIGN cells (ATCC CRL-3253) were infected with all the *Flavivirus* isolates at an MOI of 0.1, and supernatants were harvested for titration every 24 h for 6 days.

### Type I IFN Sensitivity

Vero cells were pretreated with 1, 10, and 100 U/mL of recombinant IFNα A/D (SIGMA) 16 h before infection with the *Flavivirus* isolates at an MOI of 0.1. We decided to use 1, 10, and 100 IU/mL of recombinant IFNα accordingly to studies that reported this range of concentrations in dengue patients (Kurane et al., 1993; De La Cruz Hernández et al., 2014; Talarico et al., 2017). Conversion between picograms (pg) to international units (IU) was calculated by use of a Human IFN Alpha A (Hu IFN-αA [2a]) laboratory standard calibrated to the international reference standard for Human Interferon Alpha A [Hu IFN-αA (2a)] provided by the National Institutes of Health (Meager et al., 2001).

After infection, supernatants were harvested for titration every 24 h for 6 days. The percentage of infection reduction was calculated by subtracting the titer obtained in the supernatant of preincubated Vero cells with the corresponding concentration of recombinant universal α interferon from the titer obtained in the supernatant of Vero cells infected with the corresponding *Flavivirus* isolate in the absence of recombinant α interferon multiplied by 100. *Flavivirus* isolates with a high percentage of reduction are more susceptible to the antiviral activity of recombinant α interferon. All experiments were performed in independent duplicates.

### Relative Subgenomic Flaviviral RNA Accumulation

RNA from infected HFF-1 cells with all the *Flavivirus* isolates was extracted with the miRNeasy kit (Qiagen) following the manufacturer’s recommendations. SfRNA relative accumulation was evaluated by the 2^-ΔΔCt^ method by RT-PCR with the amplification of two fragments of the flaviviral genomic gRNA. The first fragment was amplified with a gRNA forward primer, which is complementary to an upstream region of the stop codon of the open reading frame (ORF), and the gRNA reverse primer, which is complementary to the end of the 3′UTR (shared between gRNA and sfRNA). The second fragment was amplified with a sfRNA forward oligonucleotide, complementary to a region in the 3′SL in the 3′UTR, and the gRNA reverse primer. DENV sfRNA accumulation was performed with the oligonucleotides reported by Ayesa Syenina et al. (Syenina et al., 2020), and ZIKV sfRNA accumulation was performed with the following oligonucleotides: gRNA forward, 5′-ATGGTGCGCAGGATCATAGG-3′, sfRNA forward, 5′-CTGCTAGTCAGCCACAGCTT-3′, and gRNA/sfRNA reverse, 5′-CTGATCTCCAGTTCAGGCCC-3′, designed with the same rationale using the sequence of a Mexican isolate of ZIKV (GenBank KU922960.1). ΔCt values were calculated with the amplification of the housekeeping gene GADPH using oligonucleotides reported by Balm et al. (Balm et al., 2012).

### Statistical Analysis

Peak titers, the percentage of infection reduction with recombinant α interferon, and sfRNA/gRNA (2^ΔΔCt^) were compared between DENV and ZIKV isolates by two-way ANOVA to determine the effect of the virus species and between isolates from 2016 and 2019 to evaluate the effect of the epidemic year. Spearman’s correlation was assessed between hematocrit percentages and platelet counts with peak titers, percentage of infection reduction with recombinant α interferon, and 2^ΔΔCt^ of sfRNA. Finally, differences in peak titers, percentage of infection reduction with recombinant α interferon, and 2^ΔΔCt^ of sfRNA between *Flavivirus* isolates were compared with a one-way ANOVA with Bonferroni correction. Statistical analyses were done using GraphPad Prism 8.

## Results

### Isolation and Characterization of *Flavivirus* Isolated From Blood Samples From Infected Donors

**Figure 1:**
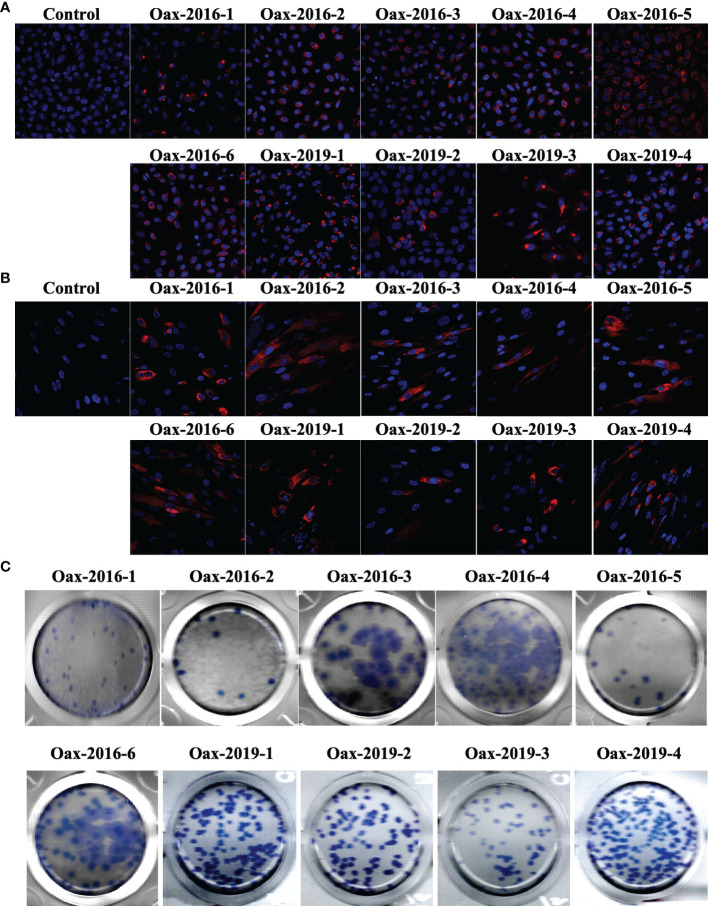
*Flavivirus* isolate identification and quantification. Immunofluorescence of Vero **(A)** and HFF-1 **(B)** cells inoculated with supernatants of blind passages of diluted sera from positive samples. Cells were fixed, permeabilized, and intracellularly stained with 4G2 monoclonal antibody followed by Alexa Fluor 546-conjugated secondary antibody. Non-inoculated cells were used as staining controls. **(C)** Plaque morphology of *Flavivirus* isolates. Supernatants of blind passages that were immunofluorescence positive were tittered in Vero cells, and plaque-forming units (PFUs) were immunostained with 4G2 monoclonal antibody followed by horseradish peroxidase (HRP)-conjugated secondary antibody. Finally, PFUs were developed with 3,3′,5,5′-tetramethylbenzidine (TMB).

We realized blind passages with diluted sera from all the samples in confluent monolayers of Vero cells. Even though all collected samples were positive for the rapid immunochromatography test, we isolated ten infectious *Flavivirus*. Initially, we confirmed the *Flavivirus* isolation by immunofluorescence. In **Figure 1**, we present confocal images of monolayers of Vero (A) and HFF-1 (B) inoculated with supernatants of blind passages of samples from infected patients and immunostained with anti-*Flavivirus* 4G2 monoclonal antibody. We observed the presence of intracellular E protein in Vero and HFF-1 cells, confirming the presence of a *Flavivirus* in the supernatant. We prepared virus stocks from immunofluorescence positive supernatants and titered them on Vero cells. In **Figure 1C**, we observe the morphology of immunostained plaques from the 10 *Flavivirus* isolates; all isolates presented round and homogenous plaque morphology except isolates Oax-2016-3, Oax-2016-4, and Oax-2016-6, which generated plaques with at least two different sizes. Finally, we identify the *Flavivirus* species by RT-PCR with RNA from infected Vero cells. All the samples were tested with the protocol of nested RT-PCR for DENV published by Lanciotti et al. (Lanciotti et al., 1992) and with the amplification of a region of NS5 of ZIKV designed by Díaz-Quiñonez et al. (Díaz-Quiñonez et al., 2016). The amplification specificity was confirmed with reference DENV and ZIKV isolates, and a representative image of the amplification pattern is presented in **Supplementary Figure 1**. The species and serotype of each isolate are listed in **Table 2**. Of the ten isolated *Flaviviruses*, four were characterized as ZIKV and six as DENV2. These results confirmed that both species cocirculate in the same epidemic year.

**Table 2:** Species and serotypes of *Flavivirus* isolates.

	ZIKV	D1-D2	Ts1	Ts2	Ts3	Ts4	Virus
**Oax-2016-1**	(+)	(−)	(−)	(−)	(−)	(−)	ZIKV
**Oax-2016-2**	(−)	(+)	(−)	(+)	(−)	(−)	DENV2
**Oax-2016-3**	(−)	(+)	(−)	(+)	(−)	(−)	DENV2
**Oax-2016-4**	(−)	(+)	(−)	(+)	(−)	(−)	DENV2
**Oax-2016-5**	(−)	(+)	(−)	(+)	(−)	(−)	DENV2
**Oax-2016-6**	(−)	(+)	(−)	(+)	(−)	(−)	DENV2
**Oax-2019-1**	(+)	(−)	(−)	(−)	(−)	(−)	ZIKV
**Oax-2019-2**	(−)	(+)	(−)	(+)	(−)	(−)	DENV2
**Oax-2019-3**	(+)	(−)	(−)	(−)	(−)	(−)	ZIKV
**Oax-2019-4**	(+)	(−)	(−)	(−)	(−)	(−)	ZIKV

Results of the RT-PCR protocols for identifying species and serotypes of the isolated Flavivirus are listed. Result for the amplification of ZIKV NS5 specific PCR product (ZIKV), DENV serotype cross-reactive PCR product (D1–D2), and serotype-specific PCR products (Ts1, Ts2, Ts3, and Ts4) are described as positive or negative accordingly for each Flavivirus isolate.

### Replication in Human Cells Varies Among *Flavivirus* Isolates From the Same Geographical Area and Year

**Figure 2:**
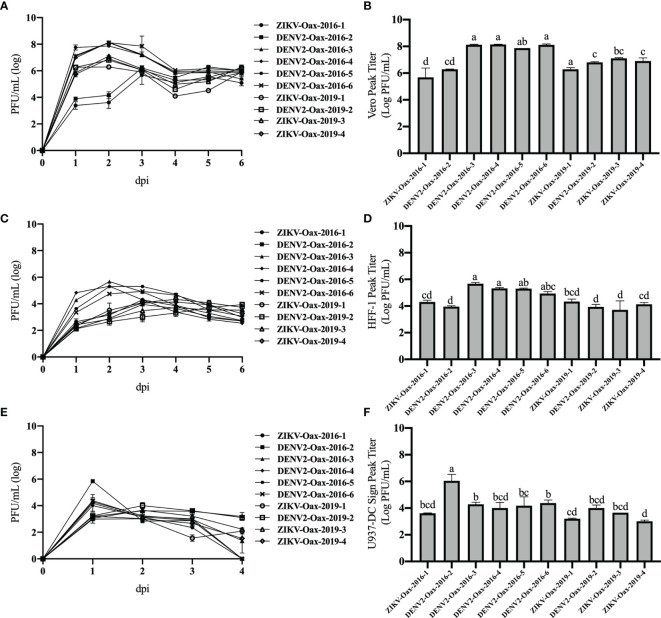
Replication curves of *Flavivirus* isolates. Multistep growth curves were performed in Vero **(A)**, HFF-1**(C)**, and U937 DC-SIGN cells **(E)** infected at a multiplicity of infection (MOI) of 0.1 with the indicated *Flavivirus* isolates and harvested from 1 to 6 days post-infection. Titers were obtained in Vero cells and are plotted on a logarithmic scale. Peak titers obtained by each isolate in Vero **(B)**, HFF-1 **(D)**, and U937 DC-SIGN cells **(F)** are presented. Letters indicate distinct groups based on the *post-hoc* statistical comparison (p < 0.05). Groups without a common letter are significantly different. Data are presented as the mean ± SD; n = 2.

Once characterized, we compared the replication curves of the ten *Flavivirus* isolates on Vero cells and the human cell lines HFF-1 and U937-DC-SIGN. We used the human dermal fibroblast cell line HFF-1 as a model to study the early stages of *Flavivirus* infection since there is evidence that replication in this skin resident cell type could represent an advantage to the virus to establish a productive infection (Montes-Gómez et al., 2020). We also evaluated replication in the human monocyte cell line U937-DC-SIGN as a model of the critical stage of *Flavivirus* infection since monocytes have been associated with the pathogenesis during DENV and ZIKV infection (Wong et al., 2012; Ayala-Nunez et al., 2019). All cell lines were infected with the *Flavivirus* isolates at an MOI of 0.1. **Figure 2** shows the multistep growth curves in Vero cells (**Figure 2A**), HFF-1 (**Figure 2C**), and U937-DC-SIGN (**Figure 2E**). From these curves, we compared the maximum titer reached by each isolate in Vero (**Figure 2B**), HFF-1 (**Figure 2D**), and U937-DC-SIGN (**Figure 2F**). The highest titers were observed in supernatants from infected Vero cells, followed by titers observed in HFF-1 cells and U937-DC-SIGN. We observed the maximum titers between 1 and 3 days post-infection.

To evaluate if there was an overall difference between virus species or epidemic years, we compared peak titers in human cell lines between DENV and ZIKV and between isolates from 2016 to 2019 by two-way ANOVA. We observed statistically significant differences in peak titers in U937-DC-SIGN between DENV and ZIKV isolates (p = 0.01867), but we did not observe differences between species in peak titers in HFF-1. In contrast, we observed statistically significant differences in peak titers in HFF-1 between isolates from 2016 and 2019 with (p = 0.03807).

To evaluate individual differences between *Flavivirus* isolates, we compared peak titers of each virus in both cell lines by one-way ANOVA with multiple comparisons and Bonferroni correction. We observed statistically significant differences between isolates from 2016 but not between isolates from 2019. Additionally, we observed statistically significant differences between isolates from 2016 and 2019 (**Figures 2D, F**). These results suggest that replication in human cells can vary between *Flavivirus* that cocirculate in the same geographical region and between epidemic years. p-Values obtained by multiple comparisons in Vero, HFF-1, and U937-DC-SIGN are discussed in detail in **Supplementary Tables 1**–**3**, respectively.

Since we observed differences in replication among *Flavivirus* isolates in human cells, we evaluated if there was a correlation between peak titer reached by DENV isolates and warning signs of dengue disease, thrombocytopenia, or elevated hematocrit percentage (WHO, 2009). We did observe a tendency to a positive correlation between peak titer in both human cell lines and hematocrit percentage and a tendency to a negative correlation between peak titers and platelet count (**Supplementary Figure 2**). However, these tendencies were not statistically significant.

### Susceptibility to the Antiviral Activity of Type I Interferon Correlates With Warning Signs in Dengue Virus-Infected Patients

Type I interferons are antiviral cytokines that limit viral replication, activating the expression of thousands of interferon-stimulated genes (ISGs) with a broad spectrum of antiviral activity. However, co-evolution with this antiviral response has selected evasion strategies in *Flavivirus* (Miorin et al., 2017). Evidence shows that the capacity to block the transcription of type I interferons genes correlates with epidemiological fitness among DENV variants (Manokaran et al., 2015). However, a comparison of susceptibility to the antiviral activity of type I interferons among *Flavivirus* isolates from the same geographical region or the same epidemic year has not been done. We compared the capability of *Flavivirus* isolates to replicate in Vero cells preincubated with 1, 10, and 100 IU/mL of recombinant universal α interferon since they are unable to secrete endogenous type I interferons but do have IFNAR and an intact JAK-STAT pathway and therefore produce ISGs in response to exogenous type I interferon stimulation (Naoki et al., 2014).

**Figure 3:**
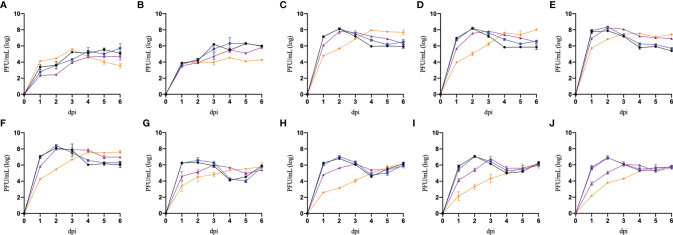
Replication curves of *Flavivirus* isolates in Vero cells preincubated with recombinant type I interferon. Vero cells were preincubated without recombinant IFNα (black lines), 1 IU/mL of recombinant universal type I interferon IFN (blue lines), 10 IU/mL of recombinant universal type I interferon IFN (purple lines), and 100 IU/mL of universal type I interferon IFN (orange lines) and infected with ten *Flavivirus* isolates: ZIKV-Oax-2016-1 **(A)**, DENV2-Oax-2016-2 **(B)**, DENV2-Oax-2016-3 **(C)**, DENV2-Oax-2016-4 **(D)**, DENV2-Oax-2016-5 **(E)**, DENV2-Oax-2016-6 **(F)**, ZIKV-Oax-2019-1 **(G)**, DENV2-Oax-2019-2 **(H)**, ZIKV-Oax-2019-3 **(I)**, and ZIKV-Oax-2019-4 **(J)** at 0.1 MOI. Supernatants of each day were collected and tittered in Vero cells; plaque-forming units (PFUs) are presented on a logarithmic scale. Data are presented as the mean ± SD; n = 2.

In **Figure 3**, we can observe that preincubation with recombinant universal α interferon significantly reduced most isolates titer, especially on the first 3 days of the replication curve. However, some isolates were able to replicate similarly in the presence of recombinant universal α interferon, suggesting differences in the susceptibility of the induced antiviral response. To compare the differences in susceptibility to the antiviral activity of recombinant universal α interferon between *Flavivirus* isolates, we calculated the percentage of infection reduction on each day of the replication curve for all *Flavivirus* isolates, normalizing the titers obtained from infected Vero cells in the presence of recombinant universal α interferon with titers without interferon.

**Figure 4:**
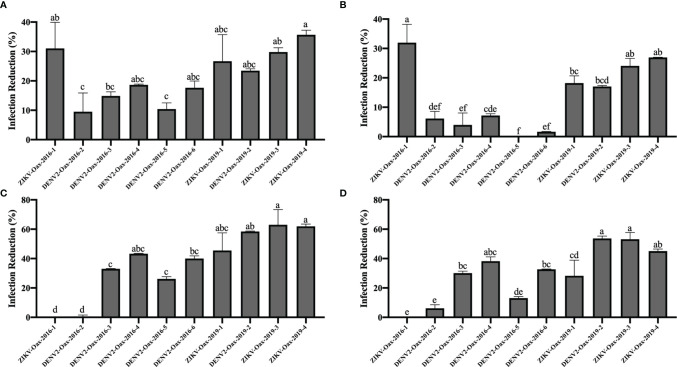
Comparison of infection reduction percentages between *Flavivirus* isolates. Percentages of infection reduction titers were calculated, normalizing the titers obtained in the presence of 10 IU/mL **(A**, **B)** and 100 IU/mL **(C**, **D)** of recombinant universal type I interferon at 1 dpi **(A**, **C)** and 2 dpi **(B**, **D)**. Letters indicate distinct groups based on the *post-hoc* statistical comparison (p < 0.05). Groups without a common letter are significantly different. Data are presented as the mean ± SD; n = 2.

We observed significant differences in the infection reduction percentages on days 1 and 2 of Vero cells preincubated with 10 and 100 IU/mL of recombinant universal α interferon; in **Figure 4**, corresponding plots of infection reduction percentages are presented. To evaluate if there was an overall difference between virus species or epidemic years, we compared infection reduction percentages between DENV and ZIKV and between isolates from 2016 to 2019 by two-way ANOVA. At 10 IU/mL of recombinant universal α interferon, we observed a statistically significant difference between virus species (1 dpi, p = 0.0009, and 2 dpi, p = 0.0001) but not between epidemic years (1 dpi, p = 0.1429, and 2 dpi, p = 0.3997). At 100 IU/mL, we also observed statistically significant differences between DENV and ZIKV (2 dpi, p = 0.0153) but also between epidemic years (1 and 2 dpi, p < 0.00001). To evaluate if there were individual differences between *Flavivirus* isolates, we compared each virus’s percentage of infection reduction by one-way ANOVA with multiple comparisons. Except for ZIKV-Oax-2016-1, *Flavivirus* isolates from 2016 were more resistant to the antiviral activity of type I interferon than isolates from 2019, especially the isolates DENV2-Oax-2016-5 and DENV2-Oax-2016-2, which presented a minimum infection reduction, even at 100 IU/mL of type I interferon (**Figures 4A–D**). The significant differences in the multiple comparisons for 10 and 100 IU/mL 1 and 2 dpi are presented in **Supplementary Tables 4**–**7**.

**Figure 5:**
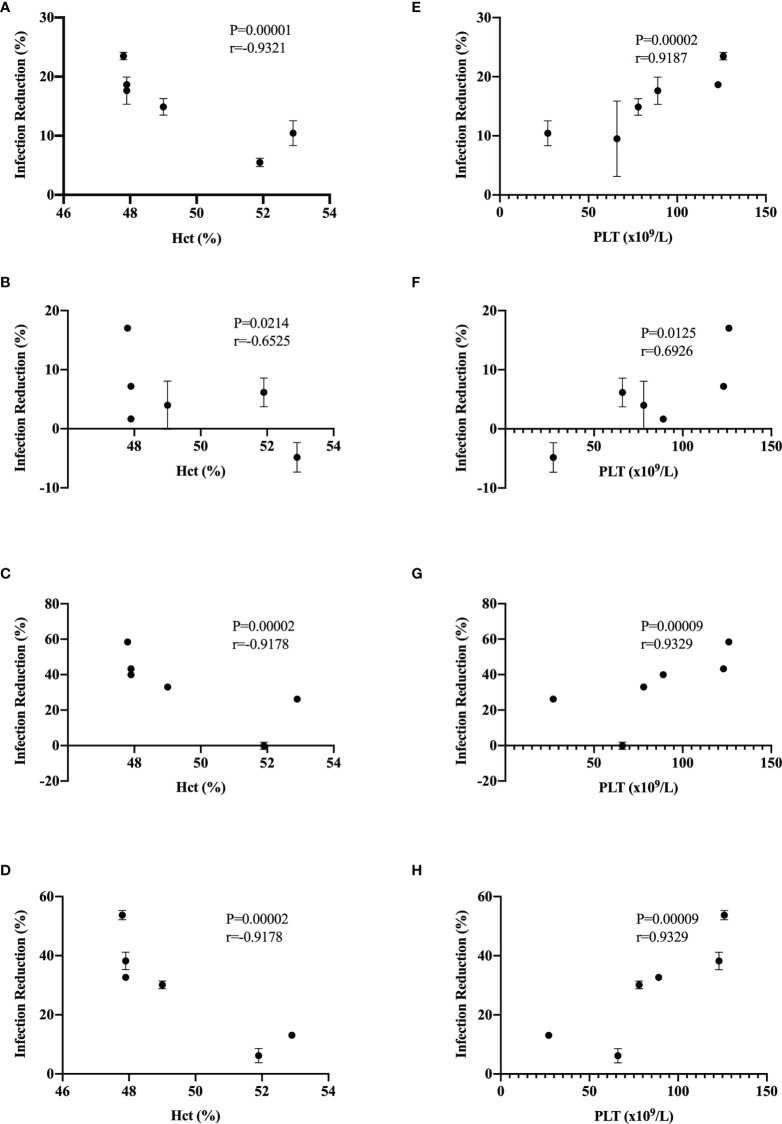
Correlation of infection reduction with warning signs. Spearman’s correlation was calculated between infection reduction percentages in Vero cells preincubated with 10 IU/mL of recombinant universal type I interferon and percentages of hematocrit at 1 dpi **(A)** and 2 dpi **(B)** and with 100 IU/mL recombinant universal type I interferon and percentages of hematocrit at 1 dpi **(C)** and 2 dpi **(D)**. Spearman’s correlation was calculated between infection reduction percentages in Vero cells preincubated with 10 IU/mL of recombinant universal type I interferon and platelet count at 1 dpi **(E)** and 2 dpi **(F)** and with 100 IU/mL of recombinant universal type I interferon and percentages of hematocrit at 1 dpi **(G)** and 2 dpi **(H)**.

Evasion of type I interferon response has been proposed as a DENV virulence factor (Green et al., 2014) and a positive fitness trait (Manokaran et al., 2015); with these antecedents, we decided to evaluate if there was a correlation between dengue disease warning signs and increment of hematocrit percentage and thrombocytopenia with type I interferon infection reduction of our DENV isolates. In **Figure 5**, we observed a significant Spearman’s correlation between the increment of hematocrit percentage (**Figures 5A–D**) and thrombocytopenia (**Figures 5E–H**) with infection reduction percentages in the presence of 10 and 100 IU/mL of recombinant universal α interferon. Our results suggest a correlation between the severity of dengue disease and evasion of the type I interferon response in *Flavivirus* isolates.

### Accumulation of Subgenomic Flaviviral RNA in Cells Infected With *Flavivirus* Isolates Correlates With Infection Reduction and Warning Signs

**Figure 6:**
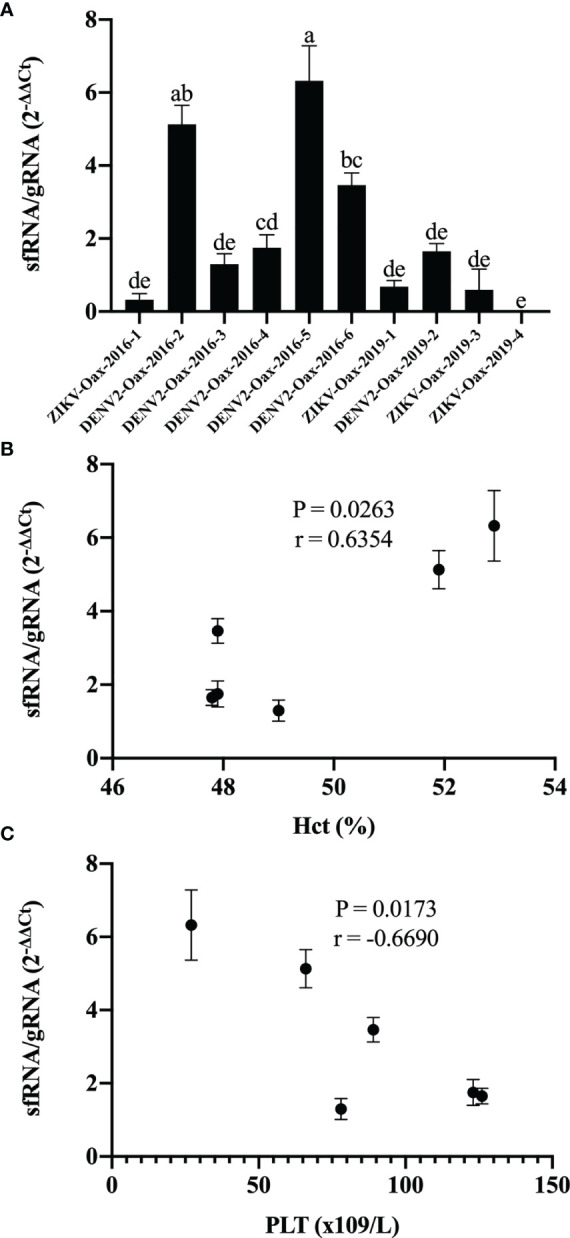
Accumulation of subgenomic flaviviral RNAs (sfRNA) in HFF-1 infected with *Flavivirus* isolates varies and correlates with infection reduction and warning signs. **(A)** RNA from HFF-1 cells infected with the *Flavivirus* isolates was used to evaluate the sfRNA relative accumulation by the 2^-ΔΔCt^ method by RT-PCR; letters indicate distinct groups based on the *post-hoc* statistical comparison (p < 0.05). Groups without a common letter are significantly different. Data are presented as the mean ± SD; n = 2. Spearman’s correlation was calculated between sfRNA/gRNA 2^−ΔΔCt^ hematocrit percentage **(B)** and platelet count **(C)**.

sfRNA play a pivotal role in evading the signaling pathways for transcription of type I interferons genes and ISGs during *Flavivirus* virus infection (Bidet et al., 2014; Manokaran et al., 2015). Since the accumulation of sfRNAs depends on the XRN1-resistant sequences in the 3′UTR, variation in this region could influence type I interferon evasion between *Flavivirus* variants. To explore this hypothesis, we infected HFF-1 cells with all the *Flavivirus* isolates and compared accumulated sfRNA by the 2^−ΔΔCt^ method by RT-PCR with the amplification of two fragments of the gRNA; the first fragment is only present in the gRNA, from the end of ORF to the end of the 3′UTR. The second fragment is shared between gRNA and sfRNA located at the end of the 3′UTR. We observed an overall difference between DENV and ZIKV in the sfRNA/gRNA (2^−ΔΔCt^) by two-way ANOVA (p = 0.0194) but no difference between epidemic years. To determine if there were differences between individual *Flavivirus* isolates, we compared the sfRNA/gRNA (2^−ΔΔCt^) by one-way ANOVA with multiple comparisons. In **Figure 6A**, we can observe that *Flavivirus* in 2016 accumulated variable amounts of sfRNA. In contrast, we did not observe differences in the sfRNA accumulation between *Flavivirus* from 2019. We also observed a correlation between sfRNA/gRNA (2^−ΔΔCt^) of DENV isolates and increment of the hematocrit percentage (p = 0263, r = 0.6354) (**Figure 6B**) and a negative correlation between sfRNA/gRNA (2^−ΔΔCt^) and platelet counts (p = 0173, r = −0.6690) (**Figure 6C**). Our results suggest that cocirculating *Flavivirus* might employ different strategies to evade the antiviral activity type I interferons.

## Discussion

*Flavivirus* infections are a public health priority in several countries, as they are linked to a wide variety of clinical manifestations ranging from asymptomatic infections to life-threatening diseases. Since the reemergence of DENV in 1978 and the introduction of Zika in 2014, Mexico has been one of the more affected countries by the cocirculation of both *Flavivirus* on an annual basis (Díaz et al., 2006; Thézé et al., 2018; Slonchak et al., 2020).

Epidemiological surveillance in Mexico mainly focuses on identifying the *Flavivirus* species and the DENV serotype that is present in every state of the country by using RT-PCR (Direccion General de Epidemiologia, 2021). Meanwhile, characterization of viral factors influencing its transmissibility and virulence of circulating variants of *Flavivirus* are limited. To study phenotypic characteristics of the circulating *Flavivirus* in Oaxaca City, one of the most affected states in the country, we isolated and compared viruses from samples of infected patients in the febrile stage of the disease from the epidemic years 2016 and 2019. We isolated ten *Flaviviruses*: four were characterized as ZIKV and six as DENV2. Although it is well known that both species cocirculate in Oaxaca, this result was surprising since according to official epidemiologic data, in 2016, 17,795 cases of DENV and 7,560 cases of Zika were confirmed; meanwhile, in 2019, 41,505 cases of DENV and only 138 cases of ZIKV were confirmed (Secretaria de Salud and Dirección General de Epidemiología). This observation alone suggests that in 2019, ZIKV was circulating more than reported. However, our sample number is too limited to support this claim.

DENV and ZIKV isolates presented similar plaque sizes except for DENV isolates DENV2-Oax-2016-3, DENV2-Oax-2016-4, and DENV2-Oax-2016-6. Traditionally, plaque size has been used as a criterion for virulence or attenuation of *Flavivirus*, and it is common to associate plaque size with *in vitro* growth rate (Liprandi, 1981). DENV isolates DENV2-Oax-2016-3, DENV2-Oax-2016-4, and DENV2-Oax-2016-6 were the *Flavivirus* isolates with the highest peak titers in Vero (**Figure 2B**) and HFF-1 (**Figure 2D**) but not in U937-DC-SIGN cells (**Figure 2F**), suggesting that *in vitro* replication rate might not be the only characteristic of the virus that determine plaque size. Recently, it has been demonstrated that plaque size also could be influenced by other variables such as evasion of the innate immune response, particularly the transcription mediated by IRF3, STAT1, and NF-κB transcription factors (Goh et al., 2016); we compared the replication of all the isolates in the presence of recombinant interferon α and DENV2-Oax-2016-3, DENV2-Oax-2016-4, and DENV2-Oax-2016-6 among the viruses with less sensitivity to the antiviral response established by α interferon (**Figure 4**), supporting the hypothesis of the influence of these phenotypes on plaque size. However, not all *Flavivirus* with high evasion phenotype presented large plaques, suggesting the multivariable effect to a simple phenotype such as plaque size.

Infection levels were observed by intracellular immunofluorescence assays (**Figure 1A**) and by replication curves in infected Vero cells (**Figures 2A, B**), human dermal fibroblasts (**Figure 2B**), and U937-DC-SIGN (**Figure 2C**), which showed differences in permissiveness among cell types; as expected, the highest peak titers were reached in Vero cells, a highly used cell line in virology because of its highest susceptibility and permissiveness to multiple viral infections, probably associated with the deletion of the type I interferon cluster on chromosome 12 (Naoki et al., 2014). We decided to infect human dermal fibroblasts since these cells are among the first permissible cell types in the skin for both DENV and ZIKV. It has been reported that infection of dermal fibroblasts induces activation of innate immune responses like secretion of IFNβ and other soluble mediators that could be key to the establishment of the antiviral and pro-inflammatory microenvironment that could shape the activation status of immune cells like dendritic cells (Kurane et al., 1992; Bustos-Arriaga et al., 2011; Hamel et al., 2015; Bustos-Arriaga et al., 2016; Kim et al., 2019). We hypothesize that *Flavivirus* isolates that efficiently infect human dermal fibroblasts could have an advantage in establishing productive infection. We observed differences in peak titers in HFF-1 between *Flavivirus* isolates from 2016 and 2019 but not between DENV and ZIKV isolates. In contrast, we did observe statistically significant differences between DENV and ZIKV isolates in U937-DC-SIGN peak titers but not between *Flavivirus* isolates from different epidemic years. Monocytes are susceptible to infection by DENV, and multiple studies have proposed their role in the severity since infected monocytes secrete high levels of pro-inflammatory cytokines (Wong et al., 2012). Monocytes have also been proven to be a target of ZIKV infection. They could play a key role in ZIKV disease since the infection has been associated with a counterbalance of monocyte/natural killer activity and increased dissemination to neural cells (Michlmayr et al., 2017; Lum et al., 2018; Ayala-Nunez et al., 2019). These observed differences between HFF-1 and U937-DC-SIGN cells could be associated with the differences in the induced innate immune response of each cell type to *Flavivirus* infection; it has been reported that DENV-infected monocytes secrete MCP-1, interferon γ-induced protein (IP)-10, IL-6, IL-8, IL-10, and IL-1β. Meanwhile, DENV- or ZIKV-infected human dermal fibroblasts secrete mainly IFNβ (Kwissa et al., 2014; Kim et al., 2019; Montes-Gómez et al., 2020). The observed differences between peak titers in dermal fibroblasts and monocytes suggest that replication efficiency varies between cocirculating *Flavivirus* variants between epidemic years.

To further explore differences in type I interferon evasion between *Flavivirus* isolates, we evaluated the replication curves in Vero cells preincubated with recombinant α interferon. Vero cells cannot secrete endogenous type I because of a homozygous deletion of approximately 9 Mb in chromosome 12. Some of the deleted genes include IFNB, IFNA8, IFNA2, IFNA1 or 13, IFNA6, and IFNA17. However, Vero cells have IFNAR and an intact JAK-STAT pathway and therefore produce ISGs in response to the type I interferon stimulation (Desmyter et al., 1968; Naoki et al., 2014). In **Figure 3**, we observe that all *Flavivirus* isolates significantly reduced their titers at 2 dpi, delaying their replication curves to reach maximum titer by at least 2 days. Several studies have correlated type I interferon concentration in infected patients with the severity of dengue and Zika disease with controversial and, in some cases, contradictory results, such as the study by Talarico et al., where they demonstrate that higher levels of IFNα or IFNβ in sera can correlate with the severity of dengue disease in pediatric patients from Paraguay (Talarico et al., 2017); in contrast, the study by De la Cruz Hernandez et al. observed that the concentration of IFNα in sera was higher in milder cases of dengue fever in comparison with samples from patients with dengue hemorrhagic fever (patients were classified according to the WHO 1997 criteria) (De La Cruz Hernández et al., 2014).

Multiple variables can influence these observed contradictions, like the phase of illness when the sample was taken, the patient’s age and health status, and even genetic background (Pichainarong et al., 2006; Maneerattanasak and Suwanbamrung, 2020; Azamor et al., 2021). Although there is evidence of variants of DENV and ZIKV that correlate with more severe clinical presentations, variability of the susceptibility to type I interferon antiviral response between circulating variants is rarely considered a factor influencing the severity of the disease. In our results presented in **Figure 4**, we demonstrate that *Flavivirus* isolated from the same geographic region and different epidemic years can present different susceptibilities to the type I antiviral response; the observed differences were as high as 30% in infection reduction between isolates from the same year (**Figure 4B**, DENV2-Oax-2016-5 vs. ZIKV-Oax-2016-1) and 63% between isolates from epidemic years at 3 years apart (**Figure 4C**, ZIKV-Oax-2016-1 vs. ZIKV-Oax-2019-4). This evidence suggests that variants with differences in the susceptibility to type I interferon antiviral response during outbreaks cocirculate and compete to dominate the ecological niche. There is no reference for the concentration range of type I interferons in the skin. However, some studies have reported a range between 10 and 100 IU/mL in sera of dengue patients. We decided to test 1, 10, and 100 IU/mL of recombinant IFNα to mimic the amount of type I interferon that the viruses would face in the infected patients (Kurane et al., 1993; De La Cruz Hernández et al., 2014; Talarico et al., 2017). We were able to observe differences in susceptibility from 10 IU/mL (**Figures 4A, B**), suggesting that the lowest concentration present in patients would be able to control replication of the susceptible isolates; however, some isolates were resistant to antiviral activity even at 100 IU/mL (**Figures 4C, D**), suggesting that some circulating variants of *Flavivirus* could replicate and establish a productive infection in patients with a high concentration of type I interferon in sera. It is interesting to observe that the isolate ZIKV-Oax-2016-1 is entirely susceptible to the antiviral response at 10 IU/mL (**Figures 4A, B**), but when the concentration is increased to 100 IU/mL, no infection reduction was observed (**Figures 4C, D**). We do not have experimental evidence to explain this observation; however, as it has been reported that some interferon-stimulated genes activated by the type I interferon signaling pathway like ISG56 can act as negative feedback regulators of the antiviral response, one possible hypothesis could be that some variants take advantage of this negative feedback regulation and gain resistance to the antiviral activity to type I interferons response after the negative regulator is expressed. Mechanistic data comparing the evasion strategies between *Flavivirus* isolates could confirm the observation that *Flavivirus* can use multiple strategies to subvert the innate immune response of the host (Li et al., 2009; Chathuranga et al., 2021).

Thrombocytopenia and increments in the hematocrit percentage in DENV-infected patients are considered warning signs by the WHO and correlate with viremia and severity of the clinical presentation of dengue (WHO, 2009; Upadhyay et al., 2017; Pathak et al., 2021). We observed a correlation of DENV isolates susceptibility to type I interferon antiviral response with the increment of hematocrit percentage and thrombocytopenia (**Figure 5**), supporting the hypothesis that type I interferon susceptibility of DENV could be a driving factor in the severity of the disease. It is possible that some observed differences in type I interferon susceptibility between isolates could be attributed to the evasion properties of sfRNAs of DENV and ZIKV; however, some isolates with low sfRNA accumulation were highly resistant to the antiviral activity of type I interferons (**Figure 4C**, ZIKV-Oax-2016-1), suggesting that other evasion strategies could be present in cocirculating *Flaviviruses* in the same epidemic event. This diversity in strategy could influence the selection of highly virulent *Flavivirus* variants.

Characterization of the transmissibility and virulence phenotype of the *Flavivirus* variants that circulate in the Mexican population could provide invaluable information on the selection dynamic as well as the variability of natural susceptibility of the hosts since available evidence suggests that genetic variability in Mexicans is particular in comparison with other Latin American populations and could influence biomedical treats (Moreno-Estrada et al., 2014). This is especially true for the Oaxaca population, as it is characterized by diverse Amerindian groups admixed with Mediterranean and African genetic backgrounds and features high diversity of HLA (González-Quezada et al., 2019). The *Flavivirus* variants that cocirculate in the Mexican population could be subjected to characteristic selection pressures; more multidisciplinary studies that could identify genetic traits that select successful *Flavivirus* variants that dominate the epidemiologic picture should be conducted.

Our study has some clear limitations starting with the limited number of isolates; however, to our knowledge, our study is the first to compare critical phenotypic traits of multiple isolates of *Flavivirus* that cocirculate in the same geographic area for two epidemic years. Additional studies of the characterization of type I interferon susceptibility among cocirculating variants of *Flavivirus* during outbreaks could explain the observed contradictions of studies correlating the type I interferon response with the severity of dengue and Zika disease and add to the knowledge of the pathogenesis of *Flavivirus*.

## Data Availability Statement

The original contributions presented in the study are included in the article/**Supplementary Material**. Further inquiries can be directed to the corresponding authors.

## Ethics Statement

The studies involving human participants were reviewed and approved by Comité de ética de la Facultad de Estudios Superiores Iztacala. Written informed consent to participate in this study was provided by the participants’ legal guardian/next of kin.

## Author Contributions

Conceived and designed the experiments: TC-J and JB-A. Recruited patients: TC-J, N-DL, HA-M, SA-R, and JB-A. Retrieved clinical data: TC-J, N-DL, HA-M, SA-R, and JB-A. Processed blood samples: TC-J, N-DL, HA-M, SA-R, and JB-A. Provided technical and scientific support and infrastructure: FA-M, FV-P, and LC-B. Performed the experiments: TC-J, LG-L, LLC, VM-T, MA-S, AP-M, JG-C, and JB-A. Analyzed the data: TC-J, JG-C, NM-D, and JB-A. Wrote the paper: TC-J and JB-A. All authors contributed to the article and approved the submitted version.

## Funding

This work was supported by Mexico’s Consejo Nacional de Ciencia y Tecnología (CONACyT) Problemas Nacionales 2016 grant number PN2029, by the UNAM PAPIIT-DGAPA programs IA209017, IA204119, and IN200821 from the National Autonomous University of Mexico (UNAM) and by Fundación Miguel Alemán A.C. for the “Estímulo a la Investigación Médica 2019.

## Conflict of Interest

Authors ND-L and HA-M were employed by company OaxacaLab Laboratorio de análisis Clínicos.

The remaining authors declare that the research was conducted in the absence of any commercial or financial relationships that could be construed as a potential conflict of interest.

## Publisher’s Note

All claims expressed in this article are solely those of the authors and do not necessarily represent those of their affiliated organizations, or those of the publisher, the editors and the reviewers. Any product that may be evaluated in this article, or claim that may be made by its manufacturer, is not guaranteed or endorsed by the publisher.
