# Supplementary material for: Variability in Susceptibility to Type I Interferon Response and Subgenomic RNA Accumulation Between Clinical Isolates of Dengue and Zika Virus From Oaxaca Mexico Correlate With Replication Efficiency in Human Cells and Disease Severity
Source: Front Cell Infect Microbiol. 2022 Jun 21;12:890750. doi: 10.3389/fcimb.2022.890750 (PMC9254156; doi:10.3389/fcimb.2022.890750)
Supplement: Supplementary file 1 [file DataSheet_1.docx]

**Supplemental Figure 1. Amplification pattern of the RT-PCR protocols.** (A) The serotype cross-reactive PCR product was amplified with the oligonucleotides D1 and D2 with RNA samples from Vero cells infected with reference strains of DENV1, DENV2, DENV4, and a DENV positive sample from Vero cells infected with Oax-2016-2 isolate. (B) Serotype-specific PCR products amplified with RNA samples from Vero cells infected with reference strains of DENV1, DENV2, and DENV4 and a DENV positive sample from Vero cells infected with Oax-2016-2 isolate. (C) ZIKV-specific PCR product was amplified with an RNA sample from Vero cells infected with a reference strain of ZIKV and the Flavivirus isolate Oax-2019-3.

**Supplemental Figure 2. Lack of correlation between warning signs and peak titer.** Spearman correlation was calculated between peak titer in HFF-1 and hematocrit percentage (A) and platelet count (B) and between peak titer in U937-DC-SIGN and hematocrit percentage (C) and platelet count (D).

|  | **Oax-2016-1** | **Oax-2016-2** | **Oax-2016-3** | **Oax-2016-4** | **Oax-2016-5** | **Oax-2016-6** | **Oax-2019-1** | **Oax-2019-2** | **Oax-2019-3** | **Oax-2019-4** |
| --- | --- | --- | --- | --- | --- | --- | --- | --- | --- | --- |
| **Oax-2016-1** |  | ns | <0. 0001= **** | <0.0001 = **** | <0. 0001= **** | <0.0001 = **** | ns | 0. 0386= * | 0. 0065= ** | 0. 0208= * |
| **Oax-2016-2** |  |  | 0. 0008= *** | 0. 0007= *** | 0. 0028= ** | 0. 0008= *** | ns | ns | ns | ns |
| **Oax-2016-3** |  |  |  | ns | ns | ns | 0. 0008= *** | 0. 0125= ** | ns | 0. 0226= * |
| **Oax-2016-4** |  |  |  |  | ns | ns | 0. 0007= *** | 0. 0108= * | ns | 0. 0194= * |
| **Oax-2016-5** |  |  |  |  |  | ns | 0.0029= ** | 0.0580= * | ns | ns |
| **Oax-2016-6** |  |  |  |  |  |  | 0.0009= *** | 0.0132= * | ns | 0.0241= * |
| **Oax-2019-1** |  |  |  |  |  |  |  | ns | ns | ns |
| **Oax-2019-2** |  |  |  |  |  |  |  |  | ns | ns |
| **Oax-2019-3** |  |  |  |  |  |  |  |  |  | ns |
| **Oax-2019-4** |  |  |  |  |  |  |  |  |  |  |

**Supplemental Table 1. Comparison of the maximum titers in Vero cells.** Maximum titers observed in the multistep growth curves were compared by one-way analysis of variance (ANOVA) with multiple comparisons and Bonferroni correction, significant differences are enlisted as follows: *P < 0.05, **P < 0.005, ***P < 0.0005, ****P < 0.00001, non-significant (ns).

|  | **Oax-2016-1** | **Oax-2016-2** | **Oax-2016-3** | **Oax-2016-4** | **Oax-2016-5** | **Oax-2016-6** | **Oax-2019-1** | **Oax-2019-2** | **Oax-2019-3** | **Oax-2019-4** |
| --- | --- | --- | --- | --- | --- | --- | --- | --- | --- | --- |
| **Oax-2016-1** |  | ns | 0.0101= * | ns | ns | ns | ns | ns | ns | ns |
| **Oax-2016-2** |  |  | 0.0016= ** | 0.0101= * | 0.0113= * | ns | ns | ns | ns | ns |
| **Oax-2016-3** |  |  |  | ns | ns | ns | 0.0127= * | 0.0015= * | 0.0005= *** | 0.004= ** |
| **Oax-2016-4** |  |  |  |  | ns | ns | ns | 0.0096= ** | 0.0028= ** | 0.0292= * |
| **Oax-2016-5** |  |  |  |  |  | ns | ns | 0.0107= * | 0.0031= ** | 0.0331= * |
| **Oax-2016-6** |  |  |  |  |  |  | ns | ns | 0.0236= * | ns |
| **Oax-2019-1** |  |  |  |  |  |  |  | ns | ns | ns |
| **Oax-2019-2** |  |  |  |  |  |  |  |  | ns | ns |
| **Oax-2019-3** |  |  |  |  |  |  |  |  |  | ns |
| **Oax-2019-4** |  |  |  |  |  |  |  |  |  |  |

**Supplemental Table 2. Comparison of the maximum titers in HFF-1 cells.** Maximum titers observed in the multistep growth curves were compared by one-way analysis of variance (ANOVA) with multiple comparisons and Bonferroni correction significant differences are enlisted as follows: *P < 0.05, **P < 0.005, ***P < 0.0005, ****P < 0.00001, non-significant (ns).

|  | **Oax-2016-1** | **Oax-2016-2** | **Oax-2016-3** | **Oax-2016-4** | **Oax-2016-5** | **Oax-2016-6** | **Oax-2019-1** | **Oax-2019-2** | **Oax-2019-3** | **Oax-2019-4** |
| --- | --- | --- | --- | --- | --- | --- | --- | --- | --- | --- |
| **Oax-2016-1** |  | <0. 0001= **** | ns | ns | ns | ns | ns | ns | ns | ns |
| **Oax-2016-2** |  |  | <0. 0001= **** | <0. 0001= **** | 0.0003= *** | <0. 0001= **** | <0. 0001= **** | <0. 0001= **** | <0. 0001= **** | <0. 0001= **** |
| **Oax-2016-3** |  |  |  | ns | ns | ns | 0.0383= * | ns | ns | 0.009= ** |
| **Oax-2016-4** |  |  |  |  | ns | ns | ns | ns | ns | ns |
| **Oax-2016-5** |  |  |  |  |  | ns | ns | ns | ns | ns |
| **Oax-2016-6** |  |  |  |  |  |  | 0.0185= * | ns | ns | 0.0045= ** |
| **Oax-2019-1** |  |  |  |  |  |  |  | ns | ns | ns |
| **Oax-2019-2** |  |  |  |  |  |  |  |  | ns | ns |
| **Oax-2019-3** |  |  |  |  |  |  |  |  |  | ns |
| **Oax-2019-4** |  |  |  |  |  |  |  |  |  |  |

**Supplemental Table 3. Comparison of the maximum titers in U937-DC-SIGN.** Maximum titers observed in the multistep growth curves were compared by one-way analysis of variance (ANOVA) with multiple comparisons and Bonferroni correction, significant differences are enlisted as follows: *P < 0.05, **P < 0.005, ***P < 0.0005, ****P < 0.00001, non-significant (ns).

|  | **Oax-2016-1** | **Oax-2016-2** | **Oax-2016-3** | **Oax-2016-4** | **Oax-2016-5** | **Oax-2016-6** | **Oax-2019-1** | **Oax-2019-2** | **Oax-2019-3** | **Oax-2019-4** |
| --- | --- | --- | --- | --- | --- | --- | --- | --- | --- | --- |
| **Oax-2016-1** |  | 0.0429 = * | ns | ns | ns | ns | ns | ns | ns | ns |
| **Oax-2016-2** |  |  | ns | ns | ns | ns | ns | ns | ns | 0.0101 = * |
| **Oax-2016-3** |  |  |  | ns | ns | ns | ns | ns | ns | ns |
| **Oax-2016-4** |  |  |  |  | ns | ns | ns | ns | ns | ns |
| **Oax-2016-5** |  |  |  |  |  | ns | ns | ns | ns | 0.0133 = * |
| **Oax-2016-6** |  |  |  |  |  |  | ns | ns | ns | ns |
| **Oax-2019-1** |  |  |  |  |  |  |  | ns | ns | ns |
| **Oax-2019-2** |  |  |  |  |  |  |  |  | ns | ns |
| **Oax-2019-3** |  |  |  |  |  |  |  |  |  | ns |
| **Oax-2019-4** |  |  |  |  |  |  |  |  |  |  |

**Supplemental Table 4. Multiple comparisons of infection reduction.** Percentages of infection reduction were compared between Flavivirus isolates in Vero cells preincubated with 10 IU/mL of recombinant type I interferon at one dpi by one-way analysis of variance (ANOVA) with multiple comparisons and Bonferroni correction, significant differences are enlisted as follows: *P < 0.05, **P < 0.005, ***P < 0.0005, ****P < 0.00001, non-significant (ns).

|  | **Oax-2016-1** | **Oax-2016-2** | **Oax-2016-3** | **Oax-2016-4** | **Oax-2016-5** | **Oax-2016-6** | **Oax-2019-1** | **Oax-2019-2** | **Oax-2019-3** | **Oax-2019-4** |
| --- | --- | --- | --- | --- | --- | --- | --- | --- | --- | --- |
| **Oax-2016-1** |  | 0.0002 = *** | <0.0001 = **** | 0.0002 = *** | <0.0001 = **** | <0.0001 = **** | 0.0301 = * | 0.0164 = * | ns | ns |
| **Oax-2016-2** |  |  | ns | ns | ns | ns | ns | ns | 0.0039 = ** | 0.0011 = ** |
| **Oax-2016-3** |  |  |  | ns | ns | ns | 0.0236 = * | 0.044 = * | 0.0015 = ** | 0.0005 = *** |
| **Oax-2016-4** |  |  |  |  | ns | ns | ns | ns | 0.0063 = ** | 0.0018 = ** |
| **Oax-2016-5** |  |  |  |  |  | ns | 0.0005 = *** | 0.0007 = *** | <0.0001 = **** | <0.0001 = **** |
| **Oax-2016-6** |  |  |  |  |  |  | 0.0074 =** | 0.0132 = * | 0.0006 = *** | 0.0002 = *** |
| **Oax-2019-1** |  |  |  |  |  |  |  | ns | ns | ns |
| **Oax-2019-2** |  |  |  |  |  |  |  |  | ns | ns |
| **Oax-2019-3** |  |  |  |  |  |  |  |  |  | ns |
| **Oax-2019-4** |  |  |  |  |  |  |  |  |  |  |

**Supplemental Table 5. Multiple comparisons of infection reduction.** Percentages of infection reduction were compared between Flavivirus isolates in Vero cells preincubated with 10 IU/mL of recombinant type I interferon at two dpi by one-way analysis of variance (ANOVA) with multiple comparisons and Bonferroni correction, significant differences are enlisted as follows: *P < 0.05, **P < 0.005, ***P < 0.0005, ****P < 0.00001, non-significant (ns).

|  | **Oax-2016-1** | **Oax-2016-2** | **Oax-2016-3** | **Oax-2016-4** | **Oax-2016-5** | **Oax-2016-6** | **Oax-2019-1** | **Oax-2019-2** | **Oax-2019-3** | **Oax-2019-4** |
| --- | --- | --- | --- | --- | --- | --- | --- | --- | --- | --- |
| **Oax-2016-1** |  | ns | 0.0031 = ** | 0.0003 = *** | 0.0193 = * | 0.0006 = *** | 0.0002 = *** | <0.0001 = **** | <0.0001 = **** | <0.0001 = **** |
| **Oax-2016-2** |  |  | 0.0037 = ** | 0.0003 = *** | 0.0233 = * | 0.0007 = *** | 0.0002 = *** | <0.0001 = **** | <0.0001 = **** | <0.0001 = **** |
| **Oax-2016-3** |  |  |  | ns | ns | ns | ns | 0.0238 = * | 0.0067 = ** | 0.0089 = ** |
| **Oax-2016-4** |  |  |  |  | ns | ns | ns | ns | ns | ns |
| **Oax-2016-5** |  |  |  |  |  | ns | ns | 0.0037 = ** | 0.0012 = ** | 0.0016= ** |
| **Oax-2016-6** |  |  |  |  |  |  | ns | ns | 0.0477 = * | ns |
| **Oax-2019-1** |  |  |  |  |  |  |  | ns | ns | ns |
| **Oax-2019-2** |  |  |  |  |  |  |  |  | ns | ns |
| **Oax-2019-3** |  |  |  |  |  |  |  |  |  | ns |
| **Oax-2019-4** |  |  |  |  |  |  |  |  |  |  |

**Supplemental Table 6. Multiple comparisons of infection reduction.** Percentages of infection reduction were compared between Flavivirus isolates in Vero cells preincubated with 100 IU/mL of recombinant type I interferon at one dpi by one-way analysis of variance (ANOVA) with multiple comparisons and Bonferroni correction, significant differences are enlisted as follows: *P < 0.05, **P < 0.005, ***P < 0.0005, ****P < 0.00001, non-significant (ns).

|  | **Oax-2016-1** | **Oax-2016-2** | **Oax-2016-3** | **Oax-2016-4** | **Oax-2016-5** | **Oax-2016-6** | **Oax-2019-1** | **Oax-2019-2** | **Oax-2019-3** | **Oax-2019-4** |
| --- | --- | --- | --- | --- | --- | --- | --- | --- | --- | --- |
| **Oax-2016-1** |  | ns | 0.0008 = *** | <0.0001 = **** | ns | 0.0004 = *** | 0.0014 = ** | <0.0001 = **** | <0.0001 = **** | <0.0001 = **** |
| **Oax-2016-2** |  |  | 0.0055 = ** | 0.0005 = *** | ns | 0.0024 = ** | 0.0103 = * | <0.0001 = **** | <0.0001 = **** | <0.0001 = **** |
| **Oax-2016-3** |  |  |  | ns | ns | ns | ns | 0.0062 = ** | 0.0074 = ** | ns |
| **Oax-2016-4** |  |  |  |  | 0.0037 = ** | ns | ns | ns | ns | ns |
| **Oax-2016-5** |  |  |  |  |  | 0.0257 = * | ns | <0.0001 = **** | <0.0001 = **** | 0.0005 = *** |
| **Oax-2016-6** |  |  |  |  |  |  | ns | 0.0034 = ** | 0.0040 = ** | ns |
| **Oax-2019-1** |  |  |  |  |  |  |  | ns | ns | ns |
| **Oax-2019-2** |  |  |  |  |  |  |  |  | ns | ns |
| **Oax-2019-3** |  |  |  |  |  |  |  |  |  | ns |
| **Oax-2019-4** |  |  |  |  |  |  |  |  |  |  |

**Supplemental Table 7. Multiple comparisons of infection reduction.** Percentages of infection reduction were compared between Flavivirus isolates in Vero cells preincubated with 100 IU/mL of recombinant type I interferon at two dpi by one-way analysis of variance (ANOVA) with multiple comparisons and Bonferroni correction, significant differences are enlisted as follows: *P < 0.05, **P < 0.005, ***P < 0.0005, ****P < 0.00001, non-significant (ns).
